# Current Evidence for a Role of Neuropeptides in the Regulation of Autophagy

**DOI:** 10.1155/2017/5856071

**Published:** 2017-05-16

**Authors:** Elisabetta Catalani, Clara De Palma, Cristiana Perrotta, Davide Cervia

**Affiliations:** ^1^Department for Innovation in Biological, Agro-Food and Forest Systems (DIBAF), Università degli Studi della Tuscia, Viterbo, Italy; ^2^Unit of Clinical Pharmacology, University Hospital “Luigi Sacco”-ASST Fatebenefratelli Sacco, Milano, Italy; ^3^Department of Biomedical and Clinical Sciences “Luigi Sacco” (DIBIC), Università degli Studi di Milano, Milano, Italy

## Abstract

Neuropeptides drive a wide diversity of biological actions and mediate multiple regulatory functions involving all organ systems. They modulate intercellular signalling in the central and peripheral nervous systems as well as the cross talk among nervous and endocrine systems. Indeed, neuropeptides can function as peptide hormones regulating physiological homeostasis (e.g., cognition, blood pressure, feeding behaviour, water balance, glucose metabolism, pain, and response to stress), neuroprotection, and immunomodulation. We aim here to describe the recent advances on the role exerted by neuropeptides in the control of autophagy and its molecular mechanisms since increasing evidence indicates that dysregulation of autophagic process is related to different pathological conditions, including neurodegeneration, metabolic disorders, and cancer.

## 1. Neuropeptides

Secretory peptides are short chains of amino acids linked together via peptide bonds which function primarily as signalling molecules in animals. In the 1970s an endogenous peptide was found in nerve cells and the term neuropeptides was then introduced [1]. After many years of intense research, there is a general agreement that neuropeptides are widely distributed throughout the central and peripheral nervous systems; they commonly act as complementary signals to “classic” neurotransmitters to fine-tune the neurotransmission, thereby controlling the balance between excitation and inhibition [2–4]. Neuropeptides may be costored or, alternatively, may coexist with other messenger molecules, as, for instance, with one or even two small classical neurotransmitters, in different cellular compartments. It is a general rule that when a peptide and a classical transmitter coexist, the former mediates long-lasting responses and the latter short-term synaptic events in the target cells. Since neuropeptides are mainly present in neurons and glial cells but are also widely expressed in nonneural cells and tissues/organs, that is, endocrine and immune systems, their functions range from neuromodulators, neurohormones/hormones, and immune-modulators to growth factors [2–7]. In this scenario, neuropeptides may act in the cross talk among nervous, endocrine, and immune systems through neurocrine, paracrine, autocrine, and endocrine manners thus influencing the postsynaptic cells and large target areas; of interest the same peptides may participate in cellular communications through different modalities. Chemically, neuropeptides have a less complex three-dimensional structure and are smaller (3–100 amino acid residues long) than normal proteins but are larger than classic neurotransmitters. More than 100 different neuropeptides are currently described in cell signalling (http://www.neuropeptides.nl).

Almost all peptidergic receptors belong to the superfamily of heterotrimeric G-protein coupled receptors (GPCRs) which are characterised by the presence of 7 transmembrane domains; but there are some exceptions, such as the ionotropic receptor for the FMRFamide and two neurotensin receptors [3–5]. Of interest, recent evidence challenges the central tenet that GPCR activity induced by neuropeptides originates exclusively at cell membrane level [8]. Commonly there are several receptor subtypes for a given peptide ligand and many naturally occurring peptides exhibit a high degree of promiscuity across GPCRs [4, 5].

## 2. Autophagy, a Brief View

Autophagy is an evolutionarily conserved membrane process involved in the replacement of cell components in both constitutive and catabolic conditions through which it plays important roles in cell functions including development, inflammation, metabolism, and aging. Autophagic process acts in a physiological manner to degrade cytoplasmic constituents, proteins, protein aggregates, and whole organelles, which are engulfed in autophagosomes which then fuse with lysosomes to form autolysosome for degradation [9, 10]. However, the role of autophagy extends beyond the general removal/recycling of damaged elements to many specific homeostatic and pathological processes [11–14].

The most prevalent form of autophagy is macroautophagy, usually simply referred to as autophagy, which is characterised by membranes that gradually grow in size to generate double membrane-structures (i.e., autophagosomes). This involves three main steps: initiation, nucleation, and expansion [9, 10, 15]. Autophagosomes recognize and sequester cellular cargo, that is, organelles, small portion of cytosol, or protein aggregates, that has been tagged by autophagy adaptors [9, 13, 15]. Cargo is then degraded by lysosomal hydrolases. Cellular cargo recognition may depend on ubiquitination, although nonubiquitinated cargo is also cleared by autophagy [16]. The molecular signalling pathway leading to autophagy is very complex and regulated by autophagy-related genes (Atgs), many of them were first identified from yeast, which are connected with the formation of autophagosomes. Atg-complexes are also controlled by several signalling pathways that fine-tune autophagy to regulate the pace of autophagosome formation. Different recent reviews have extensively reported the detailed description of the autophagic process and its regulation [9, 13, 15].

For an adequate interpretation of the data autophagy would be measured by multiple assays and monitored dynamically over time in order to assess if autophagic substrates have reached the lysosome/vacuole and whether or not they have been degraded [10, 17]. For instance, the clustering of microtubule-associated protein 1 light chain 3 (LC3) protein, a homolog of the yeast protein Atg8, and its association with autophagosomes membranes have been established as useful sign to monitor autophagy, since LC3 present in the autophagosome membrane recognizes autophagic receptors/adaptors of cargos [10, 17]. During autophagy, the cytoplasmic form of LC3-I (18 kDa) is recruited to phagophores where LC3-II (16 kDa) is generated by proteolysis and lipidation at the C-terminus. Thus LC3-II formation positively correlates with the number of autophagosomes [10, 17]. However, the lipidation and clustering of LC3 may be the result of both induction and suppression of autolysosomal maturation. In this respect, a key point in autophagy studies is that there is a difference between monitoring the autophagic elements (number or volume of autophagosomes/autolysosomes) and measuring autophagic flux during the autophagic process, as, for instance, the amount and rate of cargo sequestered and degraded [10, 17].

At the beginning autophagy was considered as a nonselective degradation mechanism, but now it is clear that selective forms of autophagy occur [10]. Depending on cell type, induction or suppression of autophagy may exert protective effects [18, 19] and altered autophagy is related to several pathologies including cancer, nervous system diseases, neurodegenerative diseases, infectious diseases, and metabolic or endocrine diseases [11–14, 20–30]. Of notice, autophagy is essential for the survival of neural cells since basal autophagy may prevent the accumulation of abnormal proteins which can disrupt neural function leading to neurodegeneration [31–33]. Autophagy is also important to accommodate the complicated architecture of neurons and their nondividing state [28]; within the endocrine system autophagy plays a critical role in controlling intracellular hormone levels, targeting both the secretory granules and the hormone-producing organelles [14].

## 3. Neuropeptidergic Systems in Autophagy

We have highlighted here recent findings that provide information on neuropeptide actions in regulating autophagy (Table 1), with an emphasis on their signalling features and pathophysiological role. Since neuropeptides are mainly present in the central nervous system but are also widely expressed and active in nonneural cells and peripheral tissues/organs, their actions have been reported in a broad spectrum of targets. This may also represent a confounding factor since neuropeptides often lack specificity at cellular levels as their signals have multiple functions.

### 3.1. Pituitary Adenylate Cyclase-Activating Polypeptide

Hypothalamic neurons are known to synthesise several neuropeptides with a variety of central and peripheral functions [34]. Among them, pituitary adenylate cyclase-activating polypeptide (PACAP) is a member of the vasoactive intestinal peptide/secretin/glucagon family of peptides. In the nervous system PACAP acts as a multifunctional peptide regulating neurotransmission, hormonal secretion, neuronal survival, neuroprotection, and neuroimmune responses [6, 35]. The peptide is also a potent antiapoptotic, anti-inflammatory, and vasodilating substance.

It has been observed that PACAP has protective effects in animal models of Parkinson disease (PD) [36], a chronic and progressive disorder which is characterised primarily by the selective loss of dopaminergic neurons in the* substantia nigra pars compacta* leading to a dopamine deficit in the striatum. Increasing evidence suggests that dysregulation of autophagy results in the accumulation of abnormal proteins and/or damaged organelles which is commonly observed in neurodegenerative diseases, including PD, although whether such dysregulation of autophagy is the cause or the consequence of PD pathology remains unclear [29, 37, 38]. LC3-II levels were found to be elevated in the* substantia nigra pars compacta* and amygdala of PD brain samples; in addition lysosomal proteins were reduced thus suggesting a link between a defect in autophagy and PD [39]. Numerous studies in both in vitro and in vivo animal models reported that the application of autophagy activators decreases dopaminergic neurodegeneration, supporting the potential therapeutic effects of autophagy modulators in PD, although other researches also report the possible harmful role of autophagy [29, 37]. Of interest, inactivation of autophagy by deleting the autophagy gene Atg7 predisposes animals to PD-like pathology [40]. Conversely, it has been recently demonstrated that the upregulation of Atg7 increases autophagy and is deleterious for dopaminergic neurons survival [41]. Products of Atg7 are essential for the activation (lipidation) of the LC3 [9, 10, 15]. In in vitro and in vivo experimental models of PD and PACAP displayed not only antiapoptotic but also antiautophagic properties since they decreased autophagic vacuole formation and lipidated LC3 levels and the expression of the autophagosomal cargo protein p62 [42], which serves as a link between LC3 and ubiquitinated substrates. PACAP also supported the correct mitochondrial function in neurons which are committed to die [42], thus suggesting its protective role during the aberrant mitophagy induced by PD.

### 3.2. Substance P

Substance P (SP) belongs to tachykinins family, which includes neuropeptides expressed in neuronal and in nonneuronal cells, as well as in noninnervated tissues [6, 43]. Among its multiple roles, SP was recently associated with increased autophagy in mouse models of chronic psychological stress condition [44]. In particular, SP increased skin levels of LC3-II and beclin-1, the mammalian orthologue of yeast Atg6 involved in autophagosome formation and maturation [9, 10, 15]. Of notice, SP was also shown to activate hyperactive bladder afferent signalling by LC3-II-mediated autophagy [45]. However, these results remain controversial since the autophagosome turnover was not investigated.

### 3.3. Agouti-Related Peptide and Proopiomelanocortin Peptides

Individual hypothalamic neuronal populations can control the body homeostasis, neuroendocrine outputs, and feeding behaviour [46]. In particular, neurons of the arcuate nucleus of the hypothalamus release specific neuropeptides that regulate feeding. Some of them increase food intake, such as orexigenic agouti-related peptide (AgRP); some others act in feeding suppression, as the anorexigenic proopiomelanocortin (POMC) synthesised by POMC neurons.

Several lines of evidence suggest a role of autophagy in the neuropeptidergic regulation of food intake and energy balance and that the regulation of hypothalamic autophagy could become an effective intervention in conditions such as obesity and the metabolic syndrome. The loss of Atg7 in AgRP neurons reduced AgRP levels, food intake (in particular refeeding response to fasting), and adiposity [47]. In contrast, deletion of Atg7 in POMC neurons increased food intake and body weight [48]. Similar results were obtained in the absence of Atg12 but not Atg5 [49]. In addition, selective loss of autophagy (i.e., loss of Atg7) in POMC neurons decreased *α*-melanocyte-stimulating hormone levels (an active derivative of POMC), increased body weight, and raised adiposity and glucose intolerance likely controlling energy balance [50, 51]. These metabolic impairments were associated with an accumulation of p62-positive aggregates in the hypothalamus and a disruption in the maturation of POMC-containing axonal projections [51]. It has been recently shown that, in hypothalamic cell lines subjected to low glucose availability, autophagy was induced via the activation of the protein kinase AMPK, which regulates the mammalian target of rapamycin (mTOR) pathway, one of the most important upstream inhibitors of the autophagic process [9], followed by decreased POMC expression [52]. Of interest the knockdown of the AMPK in the arcuate nucleus of mouse hypothalamus fed with high-fat diet decreased autophagic activity and increased POMC expression, leading to a reduction of food intake and body weight [52]. Accordingly, the impairment of POMC-derived production of adrenocorticotropin hormone was correlated with the induction of endoplasmic reticulum stress and autophagy in the pituitary glands of sucrose-rich diet-treated rats; noteworthy these effects are reversed by moderate exercise which has a beneficial role in insulin resistance [53]. Together, these data provide evidence that autophagy in POMC/AgRP neurons is required for normal metabolic regulation, neural development, and control of feeding.

### 3.4. Neuropeptide Y

Nutrient deprivation (or caloric restriction) can stimulate autophagy and the orexigenic peptide neuropeptide Y (NPY) in hypothalamic and cortical neurons [54]. NPY is one of the most abundant neuropeptides within the brain and exerts (through its receptors, named Y1 to 6) an important role in many physiological functions such as food intake, energy homeostasis, circadian rhythm, cognition, stress response, neurogenesis, and neuroprotection [6, 55–58].

In mouse hypothalamic neuronal cell line and in rat differentiated hypothalamic neural cells, NPY increased neuronal autophagic flux as shown by the analysis of LC3-II turnover, the decrease of p62, and the increase in the number of autophagosomes and autolysosomes [54]. This effect is exerted by the activation of Y1 or Y5 receptors. The signalling pathway associated with the induction of autophagy by NPY involved the activation of different protein kinases, including PI3K, ERK1/2-MAPK, and PKA. The NPY-induced autophagic flux stimulation was confirmed in mice hypothalamus by in vivo overexpression of NPY in arcuate nucleus [54]. Moreover, in rat cortical neurons NPY stimulates autophagy (i.e., the increase of LC3-II and the decrease of p62 expression) likely through the inhibition of mTOR activity [59]. In mice fed with high-fat diet, the deletion of AMPK activity in the arcuate nucleus of the hypothalamus decreased autophagy and NPY expression thus reducing food intake and body weight [52]. Accordingly, in hypothalamic cell lines, autophagy was induced via the activation of the protein kinase AMPK, modulating mTOR signalling and increasing NPY levels [52].

Since both autophagy and NPY level decrease with age, strategies to promote autophagy and increase NPY, including the caloric restriction, were suggested to produce protective effects delaying the impairments associated with longevity [54, 59, 60]. Modulating hypothalamic autophagy might have also implications for preventing obesity and metabolic syndrome of aging [47, 50]. Finally, NPY exerted a neuroprotective effect in the striatum and cerebellum of two mouse models of the spinocerebellar ataxia type 3 [61], a disease characterised by autophagic defects. Authors thus suggested that this action may be related to an activation of protein clearance mechanisms such as autophagy, even though additional data are mandatory to support this hypothesis [58, 61]. Overall, the potential of NPY to delay neurodegeneration through autophagy stimulation as a strategy to clear abnormal, misfolded proteins that cause neurodegenerative diseases deserves to be investigated in detail.

### 3.5. Ghrelin and Leptin

Ghrelin is a peptide produced primarily in the stomach and secreted into the systemic circulation. It exhibits various biological actions such as regulation of food intake, gastrointestinal motility, and energy homeostasis [62]. The adipokine leptin, the “satiety hormone,” is a peptide made by adipose cells that helps to regulate energy balance [63]. Ghrelin, the “hunger hormone,” and leptin actions are opposed. Both hormones function as neuropeptides in the hypothalamus regulating feeding.

Recent evidence suggests that ghrelin reduced mouse liver fibrosis and this event correlates with the decrease of LC3-II and an increase of p62 expression in fibrotic liver tissues [64]. Also, ghrelin promoted the cardiomyocyte survival and size maintenance during cardiac dysfunction by suppressing the excessive autophagy, as demonstrated by the decrease of LC3-II levels and autophagic vacuoles. This effect parallels the upregulation of mTOR pathway which likely acts in an AMPK-suppressed and p38-MAPK-activated manner [65]. In contrast, ghrelin stimulated insulin levels in skeletal muscles of diabetic mice, thus restoring the suppressed mTOR-dependent autophagy [66]. Accordingly, in human ovarian epithelial carcinoma cells, ghrelin inhibited mTOR, enhanced LC3-II levels, and, consequently, induced apoptosis [67]. Similarly, under caloric restriction ghrelin and NPY synergise in rat cortical neurons, stimulating autophagic flux by inhibition of mTOR [59]. Since autophagy disruption occurs in aging and age-related neurodegenerative diseases, the effects of NPY and ghrelin on autophagy activation indicate a therapeutic potential to delay aging process. In response to calorie restriction, growth hormone (GH) and liver LC3-II increased in order to maintain blood glucose level; ghrelin promotes GH secretion suggesting a mechanism for the antihypoglycaemic role of the peptide in fasted, fat-depleted mice [68].

A crucial role of autophagy was recently reported in leptin-induced proliferation of hepatic and breast cancer cells using both in vitro and xenograft models [69]. In particular, leptin caused activation of autophagy and autophagosome formation via upregulation of p53/FoxO3 axis thus favouring tumour growth and, likely, tumour invasion. In addition, the liver condition of leptin-deficient obese mice has been associated with a blockade of autophagy although data are controversial and a measurement of autophagic flux/autophagosome formation is lacking [70]. Of interest, the fact that leptin induces autophagy and acts in the pathogenesis of obesity raises the possibility of a role connecting obesity and the development of cancer caused by leptin production.

### 3.6. Somatostatin, Orexin A, and Gastrin-Releasing Peptide

Other neuropeptides are suggested to be involved in cancer initiation and progression through the modulation of autophagy. Somatostatin or somatotropin release inhibiting factor (SRIF) is a small peptide that is classically considered the key endogenous inhibitor of GH from the hypothalamus [71–76]. SRIF is present in many regions of the central and peripheral nervous systems but also in peripheral nonneuronal tissues, such as gastrointestinal tract, endocrine organs, and cells of the immune system [76–80]. Functionally, SRIF acts as neurotransmitter/neuromodulator and carries out inhibitory actions on the secretion of many biologically active substances [76, 79, 81–85]. Somatostatin analogues are the current mainstay treatment for acromegaly and gastroenteropancreatic neuroendocrine tumours [86]. It has been recently suggested that preoperative treatment with SRIF agonists of patients with acromegaly increased autophagy and decreased cell proliferation in ex vivo samples of GH-secreting adenomas [87]. In particular, SRIF treatment determined a significant decrease of immunopositivity of beclin-1 and an increase of Atg-5 staining, which is a factor inducing LC3-II and autophagosome formation [9, 10, 15].

Orexins (or hypocretins) are hypothalamic neuropeptides that regulate arousal, wakefulness, and appetite [88]. Orexin A has been shown to induce the formation of autophagic vacuoles, the lipidation of LC3-II, and the increase of beclin-1 expression in human colon cancer cells [89]. The orexin A-induced effects occurred through the upregulation of ERK pathway. In addition, the gut neuropeptide called gastrin-releasing peptide and its receptor are expressed in neuroblastoma cells and promoted angiogenesis, tumorigenesis, and metastatic potential. Noteworthy, enhanced mTOR-dependent autophagy blocked angiogenesis via degradation of gastrin-releasing peptide [90].

### 3.7. Angiotensin II

The angiogenic process and vascular endothelial status involve the role of angiotensin II (Ang-II), a peripheral hormone that increases blood pressure through vasoconstriction. Ang-II also acts as a neuropeptide in the central nervous system and is involved in neuronal dysfunction [91].

Different studies suggested that autophagy has a protective effect on vascular damage due to Ang-II since it is able to remove damaged mitochondria and other cellular organelles. For instance, in human umbilical vascular endothelial cells, Ang-II induced cell senescence and apoptosis and increased the number of autophagosomes, LC3-II, and beclin-1 expression [92]. Also, Ang-II increased autophagic flux in vascular smooth muscle cells through the production of mitochondrial reactive oxygen species [93]. In the kidney, Ang-II increased autophagosome number of podocyte and the expression of autophagic genes such as LC3-II and beclin-1, via the generation of reactive oxygen species [94, 95]. Autophagy may thus have a role also in preventing the progression of proteinuria. In cultured neonatal rat ventricular cardiomyocytes it has been reported that Ang-II-stimulated cardiomyocyte hypertrophy upregulated the expression of LC3-II as well as the number of autophagic vacuoles and the inhibition of Ang-II-induced effects on autophagy has been suggested to protect against pathological myocardial hypertrophy [96]. In this respect, it should be noted that a dual role of Ang-II has been reported in heart failure associated with autophagy modulation since some authors suggested that autophagy activation attenuated Ang-II-induced hypertrophy and vice versa [97].

### 3.8. Intermedin, Urocortin 1, and Brain Natriuretic Peptide

Intermedin (or adrenomedullin 2) is a POMC-derived neuropeptide produced by hypothalamus, pituitary, and several peripheral tissue cells with many physiological functions [98]. A role of intermedin in attenuation of myocardial infarction implicates the increase of LC3-II in a rat model of ischemic heart failure although the autophagic dynamics remains unclear [99]. Similarly, intermedin increased lipidated LC3 and autophagosome numbers in hypertrophic hearts of mice and cultured cells through the activation of both cAMP/PKA and ERK1/2-MAPK pathways, leading to the decrease in cardiomyocyte size and apoptosis [100].

Urocortin 1, a 40-amino acid peptide belonging to the corticotropin-releasing factor family, is another neuropeptide released in many areas of the brain but also in periphery including cardiac tissue [101, 102]. In particular, urocortin 1 is upregulated in the unhealthy heart and has a cardioprotective role [102, 103]. Of notice, it decreased autophagy and cell death in cardiomyocytes exposed to ischemia/reperfusion injury by reducing beclin-1 expression [104]. This effect involved the activation of PI3K/Akt signalling pathway and did not require ERK1/2-MAPK.

Brain natriuretic peptide (or ventricular natriuretic peptide) is a 32-amino acid polypeptide mainly secreted by the ventricles of the heart in response to excessive stretching of cardiomyocytes but also is present in the central nervous system where it represents an important neuromodulatory system [105]. A case report study in a 75-year-old man without overt heart failure showed augmented plasma levels of brain natriuretic peptide which may be responsible for the presence of conspicuous autophagic vacuoles in cardiomyocytes [106].

## 4. Conclusion

The current consensus is that autophagy's role as regards cell death is primarily protective [18, 19]. Indeed, in most cells, autophagy occurs at basal levels but is often increased under adverse conditions to confer stress resistance and promote cell survival, as an important cytoprotective mechanism. On the other hand high or excessive levels of autophagy may induce “autophagy cell death” [18, 19], a term used to describe cell death that is suppressed by downregulating the autophagy machine [19]. As reviewed here, recent observations, although preliminary, indicate a role for endogenous neuropeptides in the regulation of autophagy which deserves to be further investigated. This may provide a better knowledge of the molecular mechanisms and functional dynamics of autophagic process as well as its pathophysiology.

The clinical potential of neuropeptides is well known and, needless to say, the multiplicity of peptidergic receptors and the features of peptidergic transmission offer unique and important openings for the development of specific new drugs [2–7]. The study of neuropeptides in the biology of autophagy has the potential for facilitating the development of autophagy-based therapeutic interventions [107], targeting, for instance, neurodegeneration, metabolic disorders, cancer, and infection by different pathogens. For instance, urocortins and other endogenous neuropeptides such as vasoactive intestinal peptide, adrenomedullin, corticotropin-releasing hormone, ghrelin, and melanocyte-stimulating hormone have been shown to exhibit antimicrobial properties against* Trypanosoma brucei* promoting an energetic metabolism failure that triggers autophagic-like cell death [108].

The activation of autophagy may be of therapeutic benefit although there are also circumstances in which autophagic induction permits pathogenesis [18, 19]. Due to its dual pathophysiologic role, autophagy has been the subject of intensive study, in order to gain a better knowledge of its molecular mechanism and to discover new therapeutic targets. In this respect, for the treatment of autophagy-relevant human diseases, both pharmacologic activators and inhibitors of autophagic process are of interest as potential new drug candidates [30, 109, 110]. In this context, the neuropeptide system might be an exciting challenge.

## Figures and Tables

**Table 1:** Autophagy modulation of selected neuropeptides.

Compound	Role on autophagy	Targets (cell/tissue)	Effects of neuropeptides^*∗*^	Potential therapeutic applications
*PACAP*	Inhibition	Human SH-SY5Y cells; mouse brain	Decreasing apoptosis; preservation of mitochondrial activity; neuroprotection	Parkinson disease

*SP*	Activation^(?)^	Mouse skin; Wistar rat bladder	Hair cycle alteration; apoptosis	Psychological stress conditions; bladder disorders

*NPY*	Activation	Rat/mouse cortical/hypothalamic neurons; mouse hypothalamus	Neuroprotection	Control of feeding; metabolic syndrome; aging; neurodegenerative diseases

*Ghrelin*	Activation	Mouse skeletal muscle; rat cortical neurons	Restoring insulin signalling; neuroprotection	Diabetes; aging

*Ghrelin*	Inhibition	Rat H9c2 cells; human HO-8910 cells; mouse liver	Cell survival and size maintenance; reducing cell proliferation; apoptosis; decreasing expression of pathological markers	Heart failure; ovarian cancer; liver fibrosis

*Leptin*	Activation	Human HepG2 cells; human MCF-7 cells; HepG2 tumour xenografts	Tumour growth; tumour invasion; decreasing apoptosis	Obesity-associated breast and hepatic cancers

*SRIF*	Activation	Human GH-secreting adenomas	Decreasing cell proliferation	Acromegaly

*Orexin A*	Activation	Human HCT-116 cells	Decreasing cell viability	Colon cancer

*Ang-II*	Activation	Human HUVEC cells; rat vascular smooth cells; mouse podocytes; rat cardiomyocytes	Cell senescence; apoptosis; production of reactive oxygen species^§^; cardiomyocyte hypertrophy	Cardiovascular diseases; heart failure; proteinuria

*Intermedin*	Activation^(?)^	Rat H9c2 cells; mouse hearts	Attenuation of myocardial infarction; cardiomyocyte survival; improvement of cardiac performance	Heart failure; cardiac hypertrophic diseases

*Urocortin 1*	Inhibition	Rat cardiomyocytes	Decreasing apoptosis	Heart failure

^*∗*^In some cases these effects have been clearly demonstrated to be dependent on neuropeptide-induced modulation of autophagy. ^§^It has been hypothesised that autophagy has a protective effect on vascular and podocyte cell damage due to Ang-II. ^(?)^The assessment of autophagic dynamics needs further studies.
